# Potent PPARγ Ligands from *Swietenia macrophylla* Are Capable of Stimulating Glucose Uptake in Muscle Cells

**DOI:** 10.3390/molecules201219847

**Published:** 2015-12-11

**Authors:** Wai Kwan Lau, Bey Hing Goh, Habsah Abdul Kadir, Alexander Chong Shu-Chien, Tengku Sifzizul Tengku Muhammad

**Affiliations:** 1 Experimental Therapeutics Center, Institute of Pharmaceuticals and Nutraceuticals Malaysia, Blok 5-A, Halaman Bukit Gambir, Penang 11700, Malaysia; waikwan@ipharm.gov.my (W.K.L.); alex@usm.my (A.C.S.-C.); 2 Biomolecular Research Group, Biochemistry Program, Institute of Biological Sciences, Faculty of Science, University of Malaya, Kuala Lumpur 50603, Malaysia; gbh-85@hotmail.com (B.H.G.); habsah@um.edu.my (H.A.K.); 3 Biomedical Research Laboratory, Jeffrey Cheah School of Medicine and Health Sciences, Monash University Malaysia, Bandar Sunway 46150, Selangor Darul Ehsan, Malaysia; 4 School of Biological Sciences, Universiti Sains Malaysia, Penang 11800 USM, Malaysia; 5 Institute of Marine Biotechnology, Universiti Malaysia Terengganu, Kuala Terengganu 21030, Terengganu, Malaysia

**Keywords:** *Swietenia macrophylla*, PPARγ ligand, GLUT4 translocation, glucose uptake

## Abstract

Numerous documented ethnopharmacological properties have been associated with *Swietenia macrophylla* (Meliaceae), with its seed extract reported to display anti-hypoglycemic activities in diabetic rats. In the present study, three compounds isolated from the seeds of *S. macrophylla* were tested on a modified ELISA binding assay and showed to possess PPARγ ligand activity. They were corresponded to PPARγ-mediated cellular response, stimulated adipocyte differentiation but produced lower amount of fat droplets compared to a conventional anti-diabetic agent, rosiglitazone. The up-regulation of adipocytes was followed by increased adipocyte-related gene expressions such as adiponectin, adipsin, and PPARγ. The *S. macrophylla* compounds also promoted cellular glucose uptake via the translocation of GLUT4 glucose transporter.

## 1. Introduction

*Swietenia macrophylla* King (Meliaceae), commonly known as sky fruit or tunjuk langit by the locals, is a tropical timber tree native to tropical forests in America, Central America, South America and South-East Asia. Its fruit is consumed as a traditional remedy to promote blood circulation and general health. Over the years, numerous active constituents such as alkaloids, flavonoids and saponins have been extracted from *S. macrophylla* and their important ethnopharmacological properties were documented. These include anti-infective properties in pathogen, anticancer and anti-oxidation effects in cell-based and whole-organism model systems [1,2,3,4]. Besides, limonoid compounds extracted from *S. macrophylla* seeds were reported to display anti-hypoglycemic activities in diabetic rats [5,6].

Type 2 diabetes is the consequence of insufficient production or improper utilization of cellular insulin. In glucose homeostasis, insulin plays a pivotal role in the regulation of fatty acid and glucose uptake [7] by the aid of GLUT4, a glucose transporter protein. In pathophysiological conditions, peripheral tissues fail to respond to the action of insulin and consequently lead to an unwarranted decrease in the translocation of GLUT4 to the plasma membrane for glucose uptake. An activated peroxisome proliferator activated receptor γ (PPARγ), a transcription factor of a nuclear hormone superfamily, plays a crucial role in regulating GLUT4 during glucose uptake process [8], as well as in modulating transcription of genes that are involved in multiple cellular events such adipocyte differentiation, lipid storage and glucose homeostasis [9] via its ligand activation.

Thiazolidinediones (TZD) is a PPARγ agonist that possesses high binding affinity to PPARγ and has been used commercially to treat type 2 diabetes [10,11]. Despite the effectiveness of TZD as a glucose lowering drug, its utilization is limited by reports of adverse side effects, such as weight gain, fluid accumulation, liver toxicity and adverse cardiovascular effects [12]. As an alternative therapy, the search for anti-diabetic drugs has focused on the discovery of novel natural ligands for PPARγ [13]. A large number of traditional plants utilized as remedies for diabetes have been recorded [14]. An increasing number of plant-derived PPARγ ligands capable of inducing adipocyte differentiation and the expression of PPARγ and its target genes have been reported [15].

In this study, three natural compounds were isolated from *S. macrophylla* seed extract to exploit their ethnopharmacological properties, especially on the anti-hypoglycemic effect in type 2 diabetes treatment. Although numerous researches were conducted on the anti-hypoglycemic effect of *S. macrophylla* seed extract using diabetic rats, notably little is currently known regarding the underlying mechanisms that drive the glucose lowering properties of the *S. macrophylla* compounds. Therefore, this study was carried out to determine the PPARγ ligand properties of three compounds isolated from *S. macrophylla* seed extract and their transcriptional regulations, the effects of the compounds on the PPARγ-mediated cellular responses and the ability to promote cellular glucose uptake via the GLUT4 glucose transporter.

## 2. Results

### 2.1. Identification and Characterization of Compounds

**Figure 1:**
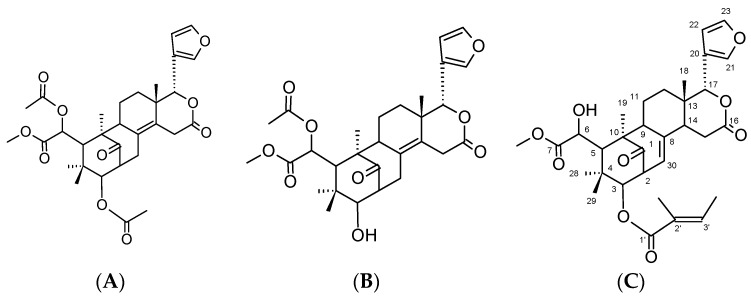
Chemical structure of the *Swietenia macrophylla* compounds. (**A**) diacetyl swietenolide; (**B**) 6-*O*-acetylswietenolide; (**C**) swietenine.

Three natural compounds namely 6-*O*-acetylswietenolide (6OA), diacetyl swietenolide (DS) and swietenine (Sw) were isolated from *S. macrophylla* seeds. The NMR spectra obtained for Sw were as follows:

Colorless needle; C_32_H_40_O_9_: EI-MS peak at *m*/*z* 568. ^1^H-NMR (CDCl_3_) δ: 3.53 (1H, ddd, *J* = 9, 8, 1.5 Hz, H-2), 4.64 (1H, d, *J* = 9 Hz, H-3), 3.50 (1H, brs, H-5), 4.56 (1H, brs, H-6), 2.30 (1H, ddd, *J* = 13, 4, 1.5 Hz, H-9), 1.81 (1H, m, H-11), 2.05 (1H, qd, *J* = 13, 4 Hz, H-11), 1.45 (1H, m, Hz, H-12), 1.74 (1H, m, H-12), 2.22 (1H, ddd, *J* = 5, 2, 1.5 Hz, H-14), 2.85 (1H, dd, *J* = 18, 5 Hz, H-15), 2.79 (1H, dd, *J* = 18, 2 Hz, H-15), 5.55 (1H, s, H-17), 0.97 (1H, s, H-18), 1.45 (1H, s, H-19), 7.55 (1H, dd, *J* = 1.8, 1 Hz, H-21), 6.38 (1H, dd, J= 1.8, 1 Hz, H-22), 7.45 (1H, t, *J* = 1.8 Hz, H-23 ), 1.12 (1H, s, H-28), 0.89 (1H, s, H-29), 5.34 (1H, dt, *J* = 8, 1.5 Hz, H-30), 3.76 (3H, s, COOCH_3_), 6.87 (1H, qd, *J* = 7, 1, 5 Hz, H-3’), 1.81 (3H, brs, 2’-CH_3_), 1.74 (3H, brd, *J* = 7, 3’-CH_3_). ^13^C-NMR δ: 216.61 (C-1), 48.93 (C-2), 78.38 (C-3), 39.01 (C-4), 45.43 (C-5), 72.84 (C-6), 175.96 (C-7), 138.27 (C-8), 57.52 (C-9), 50.36 (C-10), 21.24 (C-11), 34.60 (C-12), 36.69 (C-13), 45.04 (C-14), 29.54 (C-15), 168.48 (C-16), 76.71 (C-17), 21.24 (C-18), 16.52 (C-19), 121.33 (C-20), 140.51 (C-21), 109.21 (C-22), 143.19 (C-23), 22.77 (C-28), 23.02 (C-29), 123.60 (C-30), 53.29 (COOCH_3_), 166.91 (C-1’), 127.73 (C-2’), 139.02 (C-3’), 11.72 (2’-CH_3_), 14.63 (3’-CH_3_). The NMR spectra for the isolated Sw were in good agreement with published data [16]. DS and 6OA were isolated and separated as previously reported [17,18]. The structures of the three compounds are shown in Figure 1.

### 2.2. Binding of 6OA, DS and Sw to PPARγ in an ELISA PPARγ-Binding Assay

**Figure 2:**
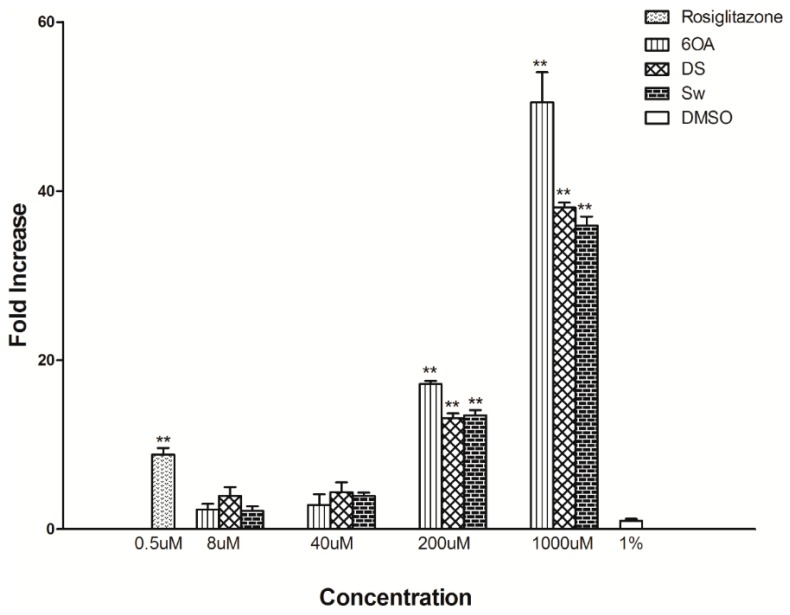
ELISA binding of PPARγ to different concentrations of the 6-*O*-acetylswietenolide (6OA), diacetyl swietenolide (DS) and swietenine (Sw) compounds isolated from *S. macrophylla* seeds. The compounds were incubated with recombinant PPARγ and bound to a 96-well plate coated with a p300 coactivator. Color development was detected through cleavage of pNPP as the substrate, and it was measured at 405 nm. The results are presented the fold increase compared with DMSO. Rosiglitazone, a known PPARγ ligand, was used as a positive control. The symbol ** denotes a significant difference at *p* < 0.01 compared with DMSO (Tukey’s test, *n* = 6).

We first evaluated the PPARγ binding properties of 6OA, DS and Sw by a modified ELISA binding assay. Results in Figure 2 revealed that the binding between PPARγ and its coactivator occurred in the presence of 6OA, DS and Sw. 200 µM and 1000 µM of compounds were found to significantly increase the binding between PPARγ and coactivator. It was also noted that 6OA produced the highest binding, with a 50-fold increase, followed by DS and Sw with 38- and 36-fold increase respectively when compared to that of vehicle (DMSO). Rosiglitazone, an anti-diabetic drug in the TZD class and a known PPARγ ligand, increased the binding between PPARγ and coactivator by 9 fold in its presence. In all cases, the concentrations of the compounds used did not produce any cytotoxicity effects with IC_50_ values of more than 100 µM on both cell types, C2C12 and 3T3-L1 cell lines.

### 2.3. Induction of 3T3-L1 Adipocyte Differentiation in 3T3-L1 Cells by 6OA, DS and Sw

**Figure 3:**
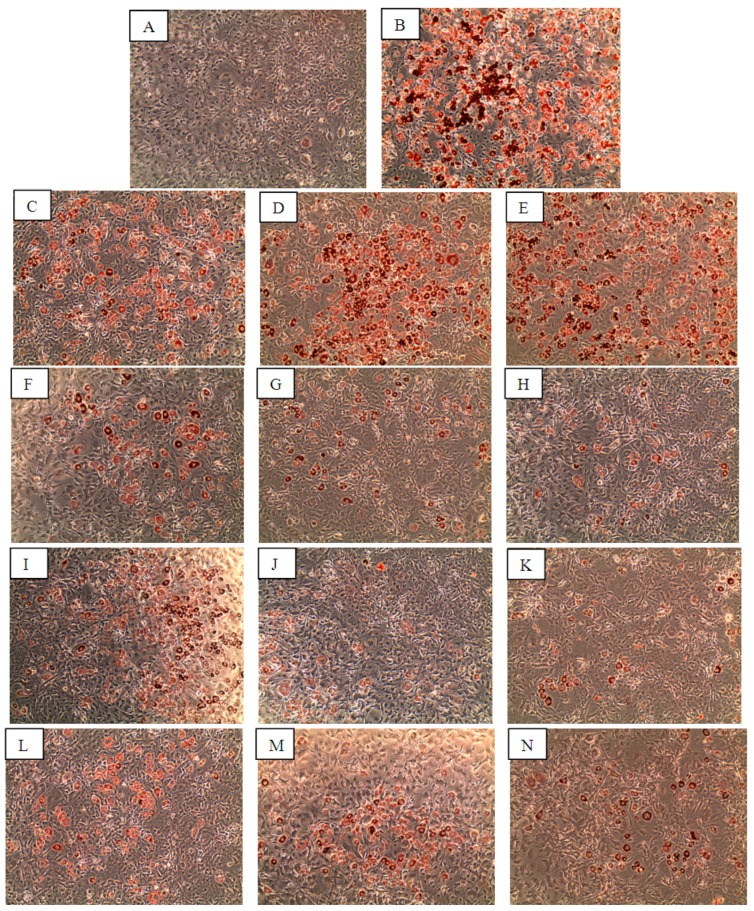
The differentiation of 3T3-L1 preadipocyte cells into mature adipocyte cells upon treatment with the compounds. The images were captured after the staining of cells treated with (**A**) DMSO; (**B**) Insulin; (**C**) 8 nM rosiglitazone; (**D**) 50 nM rosiglitazone; (**E**) 200 nM rosiglitazone; (**F**) 2 µM 6OA; (**G**) 10 µM 6OA; (**H**) 50 µM 6OA; (**I**) 2 µM DS; (**J**) 10 µM DS; (**K**) 50 µM DS; (**L**) 2 µM Sw; (**M**) 10 µM Sw and (**N**) 50 µM Sw with Oil Red O (ORO) dye 7 days after the compound treatment (*n* = 3).

**Figure 4:**
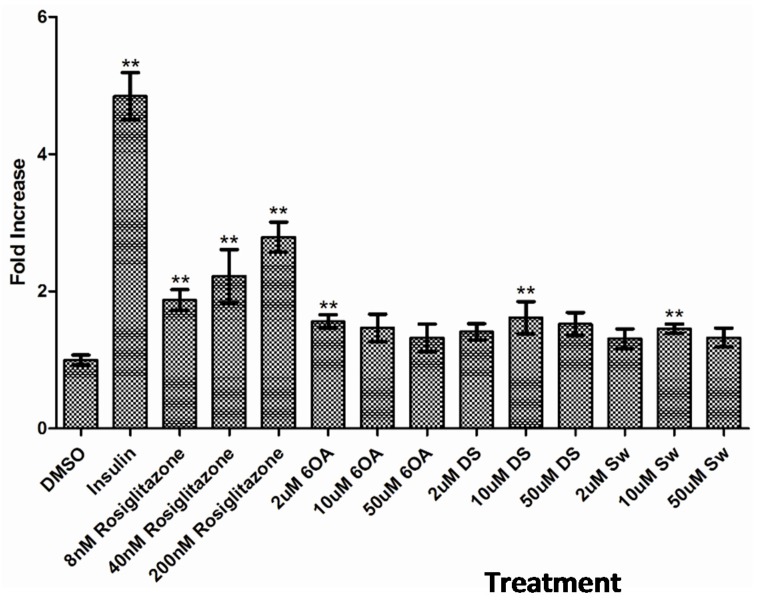
The semi-quantification of lipid droplets produced by differentiated 3T3-L1 cells at day 7 after the compound treatment. The quantification was performed by dissolving the cells in isopropanol and measuring the absorbance read out at 510 nm. The bar graph shows the relative fold increase of ORO in the cells treated with insulin, 6OA, DS and Sw compared with DMSO. The symbol ** denotes a significant difference at *p* < 0.01 compared with DMSO (Tukey’s test, *n* = 3).

Formation of lipid droplets, an activity carried out in differentiated adipocytes upon stimulation, was observed as early as day 3 after treatment with insulin; although in cells treated with the compounds or rosiglitazone, lipid droplets began appearing at day 5 of treatment. As depicted in Figure 3B–E, 3T3-L1 cells that were incubated with 1 µM insulin and a range of rosiglitazone concentrations (8–200 nM), differentiated into adipocytes which contained lipid droplets (stained red by Oil Red O (ORO)). In contrast, the cells treated with DMSO alone did not yield significant staining (Figure 3A). Compounds 6OA, DS and Sw were also shown to induce 3T3-L1 cells differentiation into lipid-producing adipocytes at 2–50 µM as shown in Figure 3F–N although at a lower level than insulin and rosiglitazone. Quantitative analysis was carried out on the ORO stained lipid droplets (Figure 4), and corresponded well with the microscopy observations. Insulin and 200 nM rosiglitazone treatments stimulated high level of lipid droplet production and the ORO stains were quantified at 4.8- and 2.8-folds higher than vehicle (DMSO), compounds 6OA, DS and Sw were also shown to stimulate lipid droplet production at 1.3–1.6-fold higher than vehicle treated cells.

### 2.4. mRNA Expression of PPARγ and Selected Adipogenic Markers in Mature Differentiated 3T3-L1 Adipocytes

**Figure 5:**
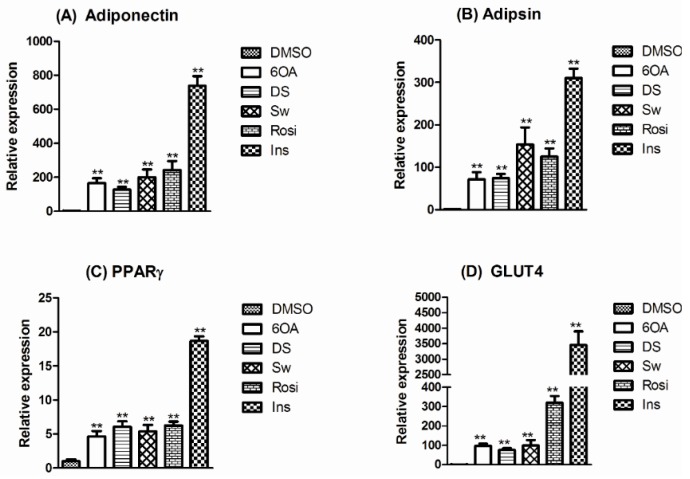
The analysis of PPARγ and selected adipogenic gene expression in differentiated 3T3-L1 adipocyte cells with quantitative RT-PCR. The expression was measured at 7 days after the treatment of cells with DMSO, 2 µM 6OA, 2 µM DS, 2 µM Sw, 50 nM rosiglitazone or 1 μM insulin. (**A**) Adiponectin; (**B**) Adipsin; (**C**) PPARγ and (**D**) GLUT4. ** denotes a significant difference compared with the basal expression from DMSO treatment at one-fold (Tukey’s test, *p* < 0.01, *n* = 3). The expression values were normalized against a housekeeping gene, β-actin.

The expression levels of selected adipogenic markers namely adiponectin, adipsin, PPARγ, and GLUT4 were studied on the three *S. macrophylla* compounds treated adipocyte cells. Insulin is a known adipocyte differentiation inducer which significantly increased the adipogenic markers’ expression as demonstrated in Figure 5. 6OA, DS, Sw as well as rosiglitazone also showed an increase in the expression levels of the adipogenic markers although at lower levels. The gene expression results correlated with the lipid accumulation studies in Section 2.3. where the expression levels of gene markers are directly proportional to the lipid droplets production.

### 2.5. GLUT4 Translocation in C2C12 Muscle Cells Treated with 6OA, DS and Sw

**Figure 6:**
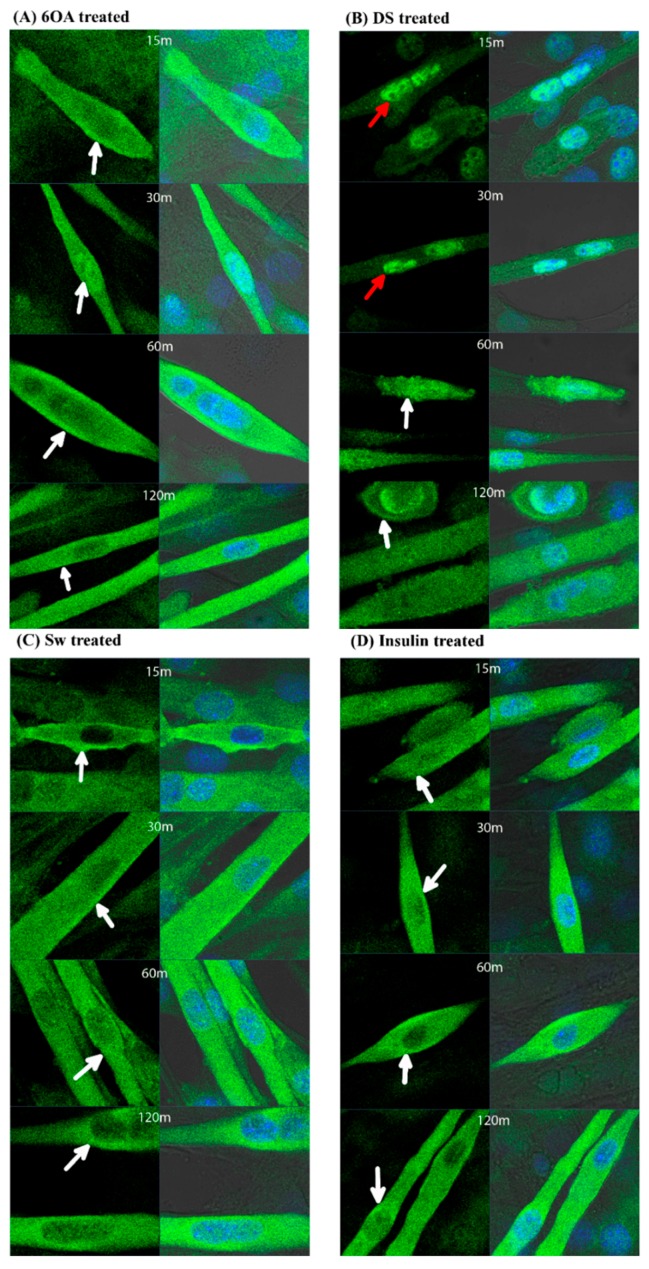

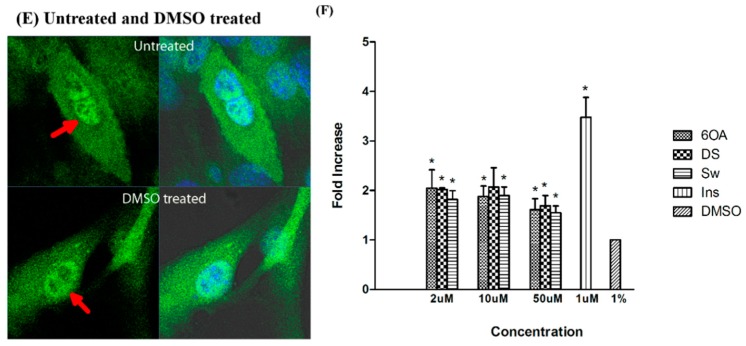
The compounds affected the distribution of GLUT4 proteins and facilitated glucose uptake in the C2C12 muscle cells. The GLUT4 proteins were visualized via the green fluorescent FITC labeled-GLUT4 antibody. The blue fluorescence shows the location of the nucleus stained by DAPI. The treatment of cells with (**A**) 2 μM 6OA; (**B**) 2 μM DS; (**C**) 2 μM Sw or (**D**) 200 nM insulin resulted in the diminished presence of GLUT4 in the nucleus and intensified presence near the plasma membrane compared with (**E**) 1% DMSO treated or untreated cells. The white arrows show the presence of GLUT4 near the plasma membrane, and the red arrows show the presence of GLUT4 within the nucleus. The figures denote the time lapse (m = minutes) after treatment. The images were captured using a LSM710 laser scanning microscope with a 63X oil immersion objective (Carl Zeiss, Oberkochen, Germany); (**F**) The amount of 2-NBDG uptake in the cells after treatment 6OA, DS and Sw or insulin was quantified at OD 510 nm. The fold increase was plotted against the treated concentration of natural compounds. The symbol * denotes a significant difference at *p* < 0.05 compared with DMSO (Tukey’s test, *n* = 6), DMSO = 1.

GLUT4 translocation from intracellular compartments to plasma membrane occurs in the presence of a stimulant. As shown in Figure 6, upon stimulation with the compounds, GLUT4 was translocated to the plasma membrane. Specifically, there was an increase in GLUT4 translocation in the C2C12 muscle cells treated with 2 µM of compounds 6OA, DS and Sw. GLUT4 translocation occurred as early as 15 min after insulin stimulation. Similar observations were found in C2C12 muscle cells treated with 6OA and Sw (Figure 6A,C). DS also resulted in GLUT4 translocation, but at a slower rate, from 60 min onwards (Figure 6B). The untreated and DMSO-treated cells depicted in Figure 6E retained most of the GLUT4 in the cytoplasm near the nucleus.

### 2.6. Glucose Uptake Assay in C2C12 Muscle Cells

The uptake of glucose in C2C12 muscle cells treated with 6OA, DS and Sw were measured using 2-NBDG, a fluorescently labelled glucose. It was shown that the treatment with 2 µM 6OA, DS or Sw produced a significant increase in glucose uptake in C2C12 muscle cells, which closely corresponded to an increase in the GLUT4 translocation to the plasma membrane. Further increase in glucose uptake was not observed when the concentration of compounds was increased to 10 µM and 50 µM, however, the levels remained significantly higher than to that of untreated cells (Figure 6F).

### 2.7. Discussion

In glucose homeostasis, insulin plays a crucial role in the regulation of a cascade of events, such as fatty acid and glucose uptake [7]. Insulin stimulates the uptake of blood glucose by adipose and muscle cells through the aid of glucose transporter proteins. The perturbed production or utilization of cellular insulin leads to the development of type 2 diabetes, which is also known as non-insulin dependent diabetes mellitus. This pathophysiological condition, mostly due to insulin resistance, is characterized by the failure of peripheral tissues to respond to the action of insulin, eventually resulting in reduced glycogen synthesis and storage in liver cells [19,20]. One possible cause of insulin resistance is an unwarranted decrease in the translocation of GLUT4, a pivotal intracellular glucose transporter, to the plasma membrane for the process of glucose uptake.

PPARγ is the key regulator of genes involved in adipocyte differentiation, lipid storage and glucose homeostasis [21]. It also plays a central role in the regulation of GLUT4 expression for the glucose uptake process [8]. Although TZD have been used commercially in the treatment of type 2 diabetes to improve insulin resistance and lower plasma glucose levels, issues related to unwanted side effects of these agents are problematic [11,22]; therefore, there is an urgent need to look into alternative natural PPARγ ligands.

*S. macrophylla* seed compounds serve as potential sources for new and safer anti-diabetic agents. In a recent study, blood glucose reduction is observed in diabetic rats that are treated with *S. macrophylla* seed extracts [6]. Besides, a compound swietenine extracted from *S. macrophylla* seeds is found to attenuate elevated cholesterol and triglyceride levels in diabetic rats [5], thus leading to the speculation of its potentials as natural oral hypoglycemic agents. Nevertheless, other bioactive constituents of *S. macrophylla* as well as their modes of action are not widely studied. Here, three *S. macrophylla* seed compounds namely 6OA, DS and Sw, were successfully isolated and their roles as PPARγ ligands were systematically reported in addition to their anti-diabetic properties. The compounds were structurally analyzed with NMR spectra and found in accordance with previous studies [16,17,18]. It is depicted in the PPARγ binding assay where recruitment of coactivator only occurred in the presence of compounds or a known synthetic PPARγ ligand, rosiglitazone. Upon binding to a natural or synthetic ligand, PPARγ is activated and subsequently triggered to regulate its downstream cellular activities. In the present study, 6OA, DS and Sw also produced corresponding gene expression patterns and cellular activities when compared with rosiglitazone. These findings strongly suggested that 6OA, DS and Sw play similar roles as the synthetic PPARγ ligand.

Adipogenesis is the process of adipocyte maturation and is typically accompanied by increased expression of adipocyte-specific genes. PPARγ is the master regulator of the adipocyte differentiation process [21] and its activation marks the activity of adipocyte differentiation. Adipogenesis is highly regulated by insulin hormone which signals to the expression of many adipocyte-specific genes. On the other hand, rosiglitazone is also capable to stimulate adipogenesis via the activation of PPARγ [8]. Compounds 6OA, DS and Sw are found to mimic the PPARγ response of rosiglitazone to initiate adipocyte differentiation, although at lower level in compared to insulin and rosiglitazone.

It is widely reported that rosiglitazone could activate PPARγ and modulate the transcription of genes involved in adipogenesis, such as adipsin and adiponectin as well as auto-activate its own gene (PPARγ) [23,24]. Our findings in Figure 4 depicted that PPARγ is elevated by five- to six folds after treated with 6OA, DS, Sw and rosiglitazone. Adiponectin is involved in the regulation of insulin sensitivity and energy homeostasis. The reduced expression and circulating adiponectin levels in obese mice and diabetic patients are improved by rosiglitazone [25]. We found that *S. macrophylla* compounds also exerted increased expression in selected adipocyte-specific genes such as adiponectin and adipsin which showed good correspondence to other gene expression studies on adipocytes induced differentiation [26,27] although to a lesser degree compared with insulin. The activation and demethylation of the GLUT4 promoter allow for GLUT4 enhancement. The up-regulation of GLUT4 expression has also characterized the presence of stimulants in adipocytes induced differentiation.

Our attention is then drawn to the downstream regulation of PPARγ in glucose homeostasis after activation as well as the possible solutions of *S. macrophylla* compounds may provide in promoting cellular glucose uptake. Skeletal muscle is one of the major tissues responding for the maintenance of whole body glucose homeostasis and accounts for 60%–70% of insulin-stimulated glucose disposal after a meal [28]. GLUT4 is a critical player in glucose homeostasis. Over-expression of GLUT4 in skeletal muscle promotes glucose uptake, which offsets insulin resistance in diabetic mice [29,30]. In our study, a widely used cell type for glucose assay uptake, skeletal muscle C2C12 cell line was utilized as the model system [31,32]. Our results showed that treating muscle cells with *S. macrophylla* compounds promoted the translocation of GLUT4 from the intracellular compartment towards the plasma membrane, and this happens as earlier as 15 min in insulin, 6OA and Sw treated cells. It is reported that the ligand activated PPARγ molecules, after undergoing conformational changes, are detached from the GLUT4 promoter and increased GLUT4 expression [8]; which shows agreement with another researchers’ works on glucose uptake reduction in the absence of PPARγ ligand is due to insufficient activity of GLUT4 [10,33]. As demonstrated by Leney and co-workers, GLUT4 is mainly sequestered in intracellular vesicles with less than 5% present on the plasma membrane and a small quantity slowly circulates between the plasma membrane and the vesicular compartment of cells. It is shown that in the presence of stimulants, more than 50% of the GLUT4 from intracellular vesicles is translocated to the plasma membrane to stimulate glucose uptake [34]. We then moved on to study the prospects of *S. macrophylla* compounds in glucose uptake stimulation and interestingly all the three compounds 6OA, DS, Sw after GLUT4 translocation induction are accompanied by an increase in glucose uptake activities.

Compounds 6OA, DS, Sw produced similar properties as known PPARγ ligands and could play an important role in mediating glucose uptake via PPARγ activation. It is tempting to speculate that they trigger the GLUT4 mechanisms to improve cellular glucose uptake. Insulin-stimulated GLUT4 expression is unalterably accompanied by excessive accumulation of adipose tissue, which is a life-threatening factor in major metabolic disorders such as obesity and diabetes [35]. Conventional PPARγ ligands like rosiglitazone, are unavoidably associated with adipogenesis and eventually leads to serious side effects [11]. On the other hand, although the *S. macrophylla* compounds possess PPARγ ligands properties and increased fair amount of adipocyte-specific genes expression, less differentiated adipocytes are yielded. Therefore, these compounds are potentiated to be high affinity PPARγ ligands in stimulating glucose uptake and reduced risk of obesity.

## 3. Experimental Section

### 3.1. Acquisition of Plant and other Chemical Materials

The seeds of *Swietenia macrophylla* King, with local name of tunjuk langit were purchased from a local market and taxonomy authenticated. A voucher specimen (No. KLU046901) was deposited at the herbarium of the Institute of Biological Sciences, University Malaya, Kuala Lumpur, Malaysia. pET28a cloning vectors were purchased from Novagen (Merck KgaA, Darmstadt, Germany) and BL21 cells were from Merck KgaA. TZD class of drug, rosiglitazone was purchased from Cayman Chemical (Ann Arbor, MI, USA). The polyclonal PPARγ IgG antibody and the alkaline phosphatase (AP) conjugated donkey anti-goat antibody were purchased from Santa Cruz (Santa Cruz, CA, USA). The anti-histidine, Tween-20 and p-nitrophenyl phosphate (pNPP) substrate were purchased from Sigma-Aldrich (Steinheim, Germany). Bovine serum albumin (BSA) was from Calbiochem (Darmstadt, Germany). Phosphate buffer saline (PBS) was purchased from Amresco (Solon, OH, USA). The microtitre 96-well plates were from Nunc (Roskilde, Denmark).

### 3.2. Construction of Recombinant Plasmids and Expression of Recombinant Proteins

The cDNA fragments of p300 and PPARγ were amplified using RT-PCR with primers designed based on the genetic sequences obtained from Genbank (Accession numbers NM_001429.1 and NM_138712.3). These cDNA fragments were cloned into a pET28a expression vector to generate recombinant plasmids with multiple histidine residues attached to their N-terminal position. The recombinant plasmids were transformed into *E. coli* BL21 cells individually to express recombinant proteins at amino acid positions 39–221 for p300, and amino acid positions 107–477 for PPARγ. The truncated p300 recombinant protein included a specific binding domain, LXXLL, for protein-protein interactions between p300 and PPARγ.

### 3.3. Preparation of Extract and Isolation of 6OA, DS and Sw from S. Macrophylla Seeds

The seeds of *S. macrophylla* were ground to a fine powder using a dry grinder, and the powder was soaked in ethanol for 3 days at room temperature. After filtration using Whatman No. 1 filter paper, the solvent was removed under reduced pressure at 45 °C using a rotary evaporator to obtain the crude ethanolic extract. The extract was further extracted repeatedly using hexane. The hexane soluble portions were combined and concentrated with a rotary evaporator. The insoluble hexane residue was partitioned using ethyl acetate and water (1:1). The *S. macrophylla* ethyl acetate fraction (SMEAF) was obtained by evaporating the soluble fraction to dryness.

A total of 3 g of the *S. macrophylla* ethyl acetate fraction was subjected to column chromatography on silica gel (70–230 mesh, 300 g). The sample was eluted with a constant gradient solvent mixture (0%–100%) with 10% increases of the chloroform proportions in *n*-hexane. Mixtures of chemical compositions were determined by thin layer chromatography (TLC). After being concentrated under reduced pressure, eleven fractions were obtained from the column chromatography. The fourth fraction (2 g) was further subjected to column chromatography (70–230 mesh, 200 g) with an initial elution using *n*-hexane followed by acetone to yield twelve fractions.

The eighth fraction (600 mg) from the previous fractionation was dissolved in methanol and stored in a refrigerator (−20 °C). After 48 h, a white solid was obtained, and a second crop was obtained after a further 48 h period. Recrystallization of the first crop (15 mg) from chloroform yielded a colorless crystal that was subsequently identified as diacetyl swietenolide (DS) (Figure 1A) [17]. Further purification of the second crop yielded 20 mg of a compound that was identified as swietenine (Sw) using spectral analysis techniques [36]. The ninth fraction (80 mg) was subjected to preparative TLC chromatography using the solvent system ethyl acetate:hexane in 1:1 ratio. The corresponding spot was extracted and recrystallized (13 mg) in chloroform. The compound was identified as 6-*O*-acetylswietenolide (6OA) [18]. The purified compounds (DS, Sw and 6OA) were subjected to all the experiments.

The structure elucidation of the Sw was determined by spectral techniques. The compound was dissolved in a CDCl_3_ solution. The ^1^H- and ^13^C-NMR spectra of the swietenine were recorded using a JEOL FT-NMR spectrometer (400 MHz). Gas chromatography-mass spectrometry (GC-MS) analysis was performed using an Agilent Technologies 6980 N gas chromatography system equipped with a 5979 Mass Selective Detector (70 eV direct inlet). An HP-5 ms (5% phenylethylsiloxane) capillary column (30.0 m × 25 mm × 25 µm) was used with helium as the carrier gas at flow rate of 1 mL/min. The column temperature was programmed initially at 150 °C, increased to 300 °C and held for 20 min at 5 °C per minute.

### 3.4. ELISA-Based Binding Assay

The ELISA binding assay used is based on the principle of binding between the ligand-dependent PPARγ and its coactivator. Briefly, 5 µg of purified recombinant p300, a PPARγ coactivator, was used to coat a 96-well microtitre plate in the presence of coating buffer (150 mM Na_2_CO_3_, 350 mM NaHCO_3_, pH 9.6). The plate was incubated for 2 h at room temperature. Nonspecific binding sites were blocked with 100 µL of blocking buffer [2% (*w*/*v*) BSA in 10 mM PBS, pH 7.2] for 2 h at room temperature. The recombinant PPARγ cell lysate was prepared by using the freeze/thaw cell lysis method [37]. A total of 100 µg of PPARγ cell lysate was incubated with 1% (*v*/*v*) DMSO (negative control), rosiglitazone (positive control) or the compound at various concentrations for 1 h at 37 °C. These mixtures were added to the p300-coated 96-well plate, which was then incubated for 1 h at 37 °C. The presence of the PPARγ ligand led to the formation of a p300-PPARγ-ligand complex. The detection of this complex was performed through the addition of 100 µL of anti-PPARγ antibody (1:1000 dilutions) and incubation for 1 h at room temperature. Subsequently, 100 µL of the AP conjugated donkey anti-goat antibody (1:1000 dilutions) was added into each well, and the plates were at room temperature for 1 h. The pNPP substrate solution (100 µL) was added to each well and incubated for 1 h at room temperature in the dark. Finally, 2.0 N sulphuric acid (50 µL per well) was added to each well to stop the enzymatic reaction. The absorbance at 405 nm was detected with a microplate reader (SpectraMax M5, Molecular Devices, Sunnyvale, CA, USA). Throughout the procedures, the microtitre plate was washed three times with washing buffer [10 mM PBS, pH 7.2, containing 0.1% (*v*/*v*) Tween 20] after each incubation step.

### 3.5. Cell Culture and Differentiation

3T3-L1 and C2C12 cells were purchased from the American Type Culture Collection (Manassas, VA, USA). The 3T3-L1 and C2C12 cells were maintained in DMEM medium supplemented with 10% (*v*/*v*) bovine serum and 10% (*v*/*v*) fetal bovine serum (FBS), respectively. The differentiation of adipocytes was initiated two days after the cells reached confluence by adding 0.5 mM isobutyl methylxanthine (IBMX), 1 µM dexamethasone and 1 µg/mL insulin to DMEM containing 10% (*v*/*v*) FBS. Two days later, the differentiation medium was replaced by DMEM containing 10% (*v*/*v*) FBS and 1 µg/mL insulin. These differentiated cells were stabilized in DMEM + 10% (*v*/*v*) FBS for another 2 days. C2C12 myocytes were induced to differentiate by substituting 10% (*v*/*v*) FBS with 10% (*v*/*v*) horse serum once the cells reached confluence. Fresh differentiation medium was replaced every two days until the myotube formation was complete.

### 3.6. Adipocytes Differentiation Assay and Gene Expression

Adipocyte differentiation is one of the major cellular responses upon PPARγ activation. 3T3-L1 pre-adipocyte cells were seeded on 24-well plates at 5 × 10^4^ cells per well. Two days after reaching confluence, the cells were triggered to differentiate by insulin (positive control), DMSO (negative control), TZD, and the three natural compounds isolated from *S. macrophylla* (6OA, DS and Sw). The medium was changed every two days until >50% of differentiated cells were observed in the positive control treated wells. For visualization, lipid droplets were stained by Oil Red O (ORO). The trapped ORO between the lipid droplets was then dissolved in absolute isopropanol, and the absorbance of the dissolved ORO was semi-quantitatively measured by a SpectraMax M5 multi-detection microplate reader (Molecular Devices) at a wavelength of 510 nm.

For the gene expression study, mature 3T3-L1 adipocytes were lysed 7 days after the induction of cellular differentiation. The total RNA samples were prepared according to the manufacturer's protocol (TRIzol reagent, Invitrogen Life Technologies, Carlsbad, CA, USA). The mRNA expression of the target genes was measured by quantitative RT-PCR with an iCycler iQ5 PCR Thermal Cycler (Bio-Rad Laboratories, Hercules, CA, USA). The primers for the adipogenesis-related genes, adiponectin, adipsin, GLUT4 and PPARγ, were designed using Primer 3 software (Table 1). Real-time PCR was performed according to the manufacturer’s protocol (SYBR-Green One-Step Quantitative RT-PCR). Briefly, 5 ng of total RNA, 50 nM of each primer, iScript RT enzyme and the universal SYBR Green reaction mix (Bio-Rad Laboratories) were mixed well in a final volume of 30 µL. All the PCR reactions were performed as follows: 1 cycle of 50 °C for 2 min and 95 °C for 10 min, followed by 40 cycles of 95 °C for 15 s and 60 °C for 1 min. The PCR products were subjected to a melting-curve analysis. The expression levels were normalized against a housekeeping gene, β-actin, and the expression levels of the genes in the DMSO treated cells were designated as 1.

**Table 1:** Primer sequences used for RT-PCR.

Gene	Direction	Sequences (5’-3’)
β-actin	Forward	TATCGCTGCGCTGGTCGTCG
	Reverse	ACAGCACAGCCTGGATGGCT
Adiponectin	Forward	TGTTGGAATGACAGGAGCTG
	Reverse	TGCTGCCGTCATAATGATTC
Adipsin	Forward	ATGACGACTCTGTGCAGGTG
	Reverse	ATTGCAAGGGTAGGGGTCTC
GLUT4	Forward	ATCCGGAACCTGGAGGGGCC
	Reverse	CGGCCAGGCCCAACAGATGG
PPARγ	Forward	GCCTGCGGAAGCCCTTTGGT
	Reverse	AAGCCTGGGCGGTCTCCACT

### 3.7. GLUT4 Translocation Assay

The differentiated C2C12 muscle cells cultured in 8-well chamber slides were treated with DMSO, insulin and *S. macrophylla* compounds for 15, 30, 60 and 120 min in DMEM low glucose + 0.1% (*w*/*v*) BSA. The cells were then fixed with 4% (*v*/*v*) paraformaldehyde, washed with MAXwash^TM^ washing medium (Active Motif, Tokyo, Japan) and blocked with Maxblock^TM^ blocking medium (Active Motif). The cells were incubated with the GLUT4 antibody for 16 h at 4 °C and then with the fluorescent conjugated secondary antibody for 2 h at room temperature. 100 µL of Fluoroshield^TM^ with DAPI (Sigma-Aldrich, St. Louis, MO, USA) was added to each well and mounted with a cover slip for imaging.

### 3.8. Glucose Uptake Assay

C2C12 cells were grown and differentiated in 96-well culture plates. The differentiated C2C12 muscle cells were treated with DMSO, insulin, and *S. macrophylla* compounds for 60 min in DMEM + 0.1% (*w*/*v*) BSA. After washing three times with Krebs-Ringer Phosphate H (KRPH) buffer + 0.1% (*w*/*v*) BSA, the cells were incubated with 100 μM 2-NBDG for 15 min. After washing three times with KRPH buffer + 0.1% (*w*/*v*) BSA, the uptake of 2-NBDG was observed under a fluorescence microscope, and the fluorescence of 2-NBDG was measured with a microplate reader (Perkin Elmer, Waltham, MA, USA).

### 3.9. Statistical Analysis

All the values were expressed as the means ±SD, and one-way ANOVA was used to determine the significance of the treatment effects at each time point. Differences among the treatment means were determined by Tukey’s test, and *p*-values were considered significant at ^∗^ *p* < 0.05, ^∗∗^ *p* < 0.01.

## 4. Conclusions

The three isolated bioactive compounds from *S. macrophylla* were demonstrated to possess the properties of PPARγ ligands. The compounds exhibited significant binding activity to PPARγ, significantly increased levels of the mRNA expression of adipogenesis-related genes and induced adipocyte differentiation, though at a low level. The compounds induced the uptake of glucose by muscle cells via an increase in the translocation of GLUT4 to the plasma membrane. *S. macrophylla* bioactive compounds exhibited a good potential for anti-diabetic activity with a minimum side effect of weight gain.
